# Cardiovascular ^18^F-fluoride positron emission tomography-magnetic resonance imaging: A comparison study

**DOI:** 10.1007/s12350-019-01962-y

**Published:** 2019-12-02

**Authors:** Jack P. M. Andrews, Gillian MacNaught, Alastair J. Moss, Mhairi K. Doris, Tania Pawade, Philip D. Adamson, Edwin J. R. van Beek, Christophe Lucatelli, Martin L. Lassen, Philip M. Robson, Zahi A. Fayad, Jacek Kwiecinski, Piotr J. Slomka, Daniel S. Berman, David E. Newby, Marc R. Dweck

**Affiliations:** 1 British Heart Foundation Centre of Cardiovascular Sciences, University of Edinburgh, Room SU.305, Chancellor’s building, 51 Little France Crescent, University of Edinburgh, Edinburgh, EH16 4SB UK; 2 Edinburgh Imaging, Queen’s Medical Research Institute, University of Edinburgh, Edinburgh, UK; 3 Christchurch Heart Institute, University of Otago, Christchurch, New Zealand; 4 Cedars Sinai Medical Center, Los Angeles, CA USA; 5 Icahn School of Medicine at Mount Sinai, New York, PA USA

**Keywords:** PET, PET/MR, PET/CT, atherothrombosis, aortic stenosis, myocardial infarction, CMR

## Abstract

**Background:**

^18^F-Fluoride uptake denotes calcification activity in aortic stenosis and atherosclerosis. While PET/MR has several advantages over PET/CT, attenuation correction of PET/MR data is challenging, limiting cardiovascular application. We compared PET/MR and PET/CT assessments of ^18^F-fluoride uptake in the aortic valve and coronary arteries.

**Methods and results:**

18 patients with aortic stenosis or recent myocardial infarction underwent ^18^F-fluoride PET/CT followed immediately by PET/MR. Valve and coronary ^18^F-fluoride uptake were evaluated independently. Both standard (Dixon) and novel radial GRE) MR attenuation correction (AC) maps were validated against PET/CT with results expressed as tissue-to-background ratios (TBRs). Visually, aortic valve ^18^F-fluoride uptake was similar on PET/CT and PET/MR. TBR_MAX_ values were comparable with radial GRE AC (PET/CT 1.55±0.33 *vs.* PET/MR 1.58 ± 0.34, *P* = 0.66; 95% limits of agreement − 27% to + 25%) but performed less well with Dixon AC (1.38 ± 0.44, *P* = 0.06; bias (−)14%; 95% limits of agreement − 25% to + 53%). In native coronaries, ^18^F-fluoride uptake was similar on PET/MR to PET/CT regardless of AC approach. PET/MR identified 28/29 plaques identified on PET/CT; however, stents caused artifact on PET/MR making assessment of ^18^F-fluoride uptake challenging.

**Conclusion:**

Cardiovascular PET/MR demonstrates good visual and quantitative agreement with PET/CT. However, PET/MR is hampered by stent-related artifacts currently limiting clinical application.

**Electronic supplementary material:**

The online version of this article (10.1007/s12350-019-01962-y) contains supplementary material, which is available to authorized users.

## Introduction

Calcification is a key pathological process in both aortic stenosis and coronary atherosclerosis. The development of ^18^F-fluoride imaging now allows calcification activity to be imaged directly, providing a marker of disease activity in aortic stenosis and coronary atherosclerosis with potential to improve patient risk stratification.

To date, cardiac studies investigating the uptake of ^18^F-fluoride have predominantly employed hybrid PET/CT, but interest has recently developed in Positron Emission Tomography/Magnetic Resonance (PET/MR) platforms. PET/MR provides several potential advantages compared to PET/CT including reduced radiation exposure, integrated functional assessment, improved soft tissue characterisation and motion correction.^1^ While PET/MR has already shown promise in the assessment of aortic stenosis,^2^ coronary atherosclerosis,^3^ cardiac amyloidosis and sarcoidosis,^4^,^5^ it remains unclear how the pattern and intensity of tracer uptake compares with the current gold-standard of PET/CT. This is of particular importance given concerns regarding the optimal method for attenuation correction on PET/MR.

MR-based attenuation correction maps are based on proton density,^6^ with two current approaches. The Dixon MR AC map is the standard approach but is hampered in cardiac studies due to both motion artifact (along the heart–lung and liver–lung interfaces) and mis-segmentation of the bronchi as soft tissue. A novel free-breathing radial GRE (Gradient Recalled Echo) approach was developed to try to overcome these issues.^3^ Concerns remain for both techniques about the impact of intra-coronary stents on attenuation correction.

We aimed to validate PET/MR (using both the Dixon and radial GRE attenuation correction approaches) against PET/CT in patients undergoing the two paired scans. In particular, we sought to compare the pattern of aortic valve and coronary ^18^F-fluoride uptake on PET/MR and PET/CT and to investigate whether quantification of tracer uptake in these regions differs between the two imaging approaches.

## Methods

Study subjects from two ongoing ^18^F-fluoride PET/CT trials were approached regarding participation in the current study and having a PET/MR scan immediately following their PET/CT. The SALTIRE 2 trial (Bisphosphonates and RANKL Inhibition in Aortic Stenosis, NCT02132026) recruited patients aged over 50 years with a peak aortic jet velocity of >2.5 m/s and grade 2-4 calcification of the aortic valve on echocardiography. The PRE^18^FFIR trial (Prediction of recurrent events with ^18^F-fluoride to identify ruptured and high-risk coronary artery plaques in patients with myocardial infarction, NCT02278211) recruited patients with recent myocardial infarction and multi-vessel coronary artery disease on invasive angiography. Exclusion criteria for both trials include inability to receive iodinated contrast, renal impairment (estimated glomerular filtration rate ≤30 mL/min/1.73 m^2^) or women of child-bearing potential. This PET/CT and PET/MR comparison study was approved by the Scottish Research Ethics Committee and the United Kingdom (UK) Administration of Radiation Substances Advisory Committee. It was performed in accordance with the Declaration of Helsinki. All patients provided written informed consent prior to any study procedures.

### ^18^F-Fluoride Positron Emission Tomography and Coronary Computed Tomography Angiography

All patients underwent a single ^18^F-fluoride PET/CT scan. Patients were administered 50-100 mg oral metoprolol if their resting heart rate was >65 beats/min prior to the intravenous administration of 125 MBq ^18^F-fluoride (aortic stenosis cohort) or 250 MBq ^18^F-fluoride (myocardial infarction cohort). After 60 minutes, patients were imaged with a hybrid PET/CT scanner (64-multidetector Biograph mCT, Siemens Healthcare GmbH, Erlangen, Germany). CT attenuation correction scans were performed as follows; Helical and on inspiration at 120 kV with mA adjusted to body habitus. The pitch was 0.8, slice thickness 5 mm rotation time 500 ms. PET-emission scans were then acquired in list-mode format (30 minutes).

All patients in the PRE^18^FFIR myocardial infarction cohort received sublingual glyceryl trinitrate prior to CCTA. CT effective dose was calculated by multiplying the dose length product (mGy · cm) by a conversion factor (0.014 mSv/mGy · cm).

### ^18^F-Fluoride Positron Emission Tomography and Coronary Magnetic Resonance Angiography

Immediately after the PET/CT scan all participants were transferred onto the hybrid PET/MR system (Biograph mMR, Siemens Healthcare GmbH, Erlangen, Germany) for simultaneous ^18^F-fluoride PET/MR imaging. PET data were acquired for 50 minutes in list-mode, starting approximately 120 minutes after intravenous injection of ^18^F-fluoride. Both standard breath-held 3D Dixon-VIBE^7^ (Dixon) and free-breathing radial gradient echo (GRE, Siemens work-in-progress #793F) sequences were acquired for MR attenuation correction with the subjects arms down (HUGE sequence not employed). The free-breathing radial GRE attenuation correction map was generated during PET acquisition (for 4 minutes 52 seconds) using the method described by Robson et al^6^ The MR protocol also included coronary magnetic resonance angiography (CMRA) performed with 0.2 mmol/kg of intravenous gadobutrol contrast (Gadovist, Bayer Pharma AG, Germany) and late gadolinium enhancement imaging 10-15 minutes post contrast administration. The total MRI scan duration was approximately 60 minutes.

### Image Reconstruction

Both PET/CT and PET/MR off-line PET reconstructions were carried out using e7tools (Siemens Healthcare). The full list-mode acquisitions were reconstructed without time-of-flight correction or resolution modeling. Ordered Subsets Expectation Maximization (OSEM) algorithm with the following parameters were employed: 256 × 256 field of view, 4 iterations, 21 subsets, 5 mm Gaussian filter. The CT was used for attenuation correction of the PET/CT data. PET data acquired on PET/MR were first reconstructed applying the standard Dixon attenuation correction method (4 tissue class segmentation; air, lung, soft tissue and fat). PET data were then also reconstructed applying a custom MR attenuation correction map derived from the free-breathing radial GRE sequence [2 tissue classes: background (air and lung) and soft tissue (soft tissue and fat)].^3^,^8^ ECG gating was not applied for either modality.

### PET/CT and PET/MR Image Analysis

**Figure 1:**
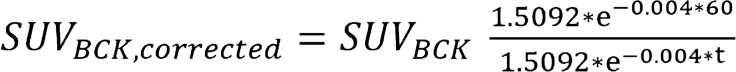
Correction factor formula to compensate for variations in injection-to-scan interval. ‘*t*’ represents tracer circulating time prior to PET imaging

Accurate co-registration was achieved by aligning ^18^F-fluoride activity in the blood pool and ascending aorta with the corresponding anatomical structures on the CCTA.^9^ Qualitative and semi-quantitative analysis of the PET images from all 18 scans was performed independently by a trained observer (J.P.M.A.) using FusionQuant software (Version 1, Cedars-Sinai Medical Center, Los Angeles, USA). Radiotracer uptake was analyzed using a standardized protocol (Supplemental data). For aortic valve analysis, polygons of 6-mm depth were drawn around the perimeter of the valve on the co-registered co-axial image to generate a region of interest (ROI) (Figure 1C, H+M yellow dotted line).^10^ Coronary arteries with a diameter ≥ 2 mm were assessed according to the 18-segment Society of Cardiac Computed Tomography model.^11^ Coronary uptake was considered positive if an area of increased activity originated in a diseased coronary artery and followed its course for > 5 mm in 3 dimensions across orthogonal views.^12^ Standardized uptake values (SUV_MAX_) were calculated for all ROIs and corrected for blood pool activity (measured in the right atrium^13^) to generate tissue-to-background ratios (TBR_MAX_).

A previous study evaluating the diagnostic effect of a prolonged circulation time on coronary uptake, established that imaging at later time points following tracer injections did not affect SUV values but increased TBR values predominantly as a result of increased tracer clearance from the blood pool.^12^ We therefore applied a previously validated correction factor that compensates for this effect and the fact that patients were imaged at a later time point with PET/MR than PET/CT.^14^ This approach individually corrects all SUV measurements of blood pool activity (accounting for transfer time between scanners) to a standard 60-min time point post-injection, thereby correcting our calculated PET/CT and PET/MR TBR values for differences in injection-to-scan time (Suppl. Figure 1).

### Statistical Analysis

Statistical analyses were performed using GraphPad Prism Version 7.0 and SPSS Version 23. A two-sided *P* < 0.05 was considered statistically significant. The distribution of all continuous variables was assessed using the D’Agostino and Pearson test, which were presented using mean ± standard deviation of the mean or median [interquartile range]. Comparisons between groups were performed using the two-sample *t* test, one-way ANOVA (paired where appropriate), Bland–Altman method of comparison, intra-class correlation coefficient (with 95% confidence intervals for continuous measurements) and Kappa statistic where appropriate. All categorical variables are presented as percentages.

## Results

### Patients

A total of 18 patients (mean age 67 ± 7 years, 16 male; Table 1) were recruited and completed both PET/CT and PET/MR scans of the aortic valve and coronary arteries: 7 with aortic stenosis and 11 with recent myocardial infarction. There was no difference in net PET counts on the 30 minutes PET/CT compared to the 50 minutes PET/MR (*P* = 0.66). CT exposure added 4.3 ± 1.2 mSv (49%) to the overall total effective radiation dose (8.8 mSv).

**Table 1:** Participant demographics

	Whole cohort (*n* = 18)	Aortic Stenosis (*n* = 7)	Myocardial Infarction (*n* = 11)
Age	67 (56–78)	69 (57–78)	67 (59–76)
Male	16/18 (89%)	6/7 (86%)	10/11 (91%)
Smoking (ex or current)	5/18 (28%)	1/7 (14%)	4/11 (36%)
Hypertension	7/18 (39%)	3/7 (43%)	4/11 (36%)
Hyperlipidaemia	10/18 (56%)	4/7 (57%)	6/11 (55%)
Diabetes	1/18 (6%)	1/7 (14%)	0/11 (0%)
Previous myocardial Infarction	12/18 (67%)	1/7 (14%)	11/11 (100%)
Previous PCI	13/18 (72%)	2/7 (29%)	11/11 (100%)
Administered dose ^18^F-Fluoride (MBq)	193.7 ± 61.5	119.4 ± 6.2	241.6 ± 8.0
PET effective radiation dose (mSv)	4.5	3	6
CT Dose Length Product (mGy/cm)	310.1 ± 89.3	336.1 ± 32.9	293.5 ± 110.1
CT effective radiation dose (mSv)	4.3 ± 1.2	4.7 ± 0.5	4.1 ± 1.5
PET/CT injection-to-scan interval (mins)	62 ± 5	61 ± 2	63 ± 5
PET/MR injection-to-scan interval (mins)	136 ± 16	123 ± 5	143 ± 18

*CT*, Computerized Tomography; *MBq*, megabecquerels; *mGy/cm*, milligrays/centimeter, *mins*, minutes; *mSv*, millisievert; *PCI*, Percutaneous Coronary Intervention; *PET/MR*, Positron Emission Tomography/Magnetic Resonance

### Qualitative Image Quality

When the standard Dixon AC technique was used, substantial extra-cardiac artifact affected PET/MR image interpretation in all 18 study participants. These consisted of increased tracer activity in the bronchial tree and at the heart-lung and liver interfaces as previously described.^3^ The free-breathing radial GRE attenuation correction map eliminated these artifacts on all the PET/MR scans (Suppl. Figure 2). None of these extra-cardiac artifacts were present on the PET/CT scans.

### Aortic Stenosis

**Figure 2:**
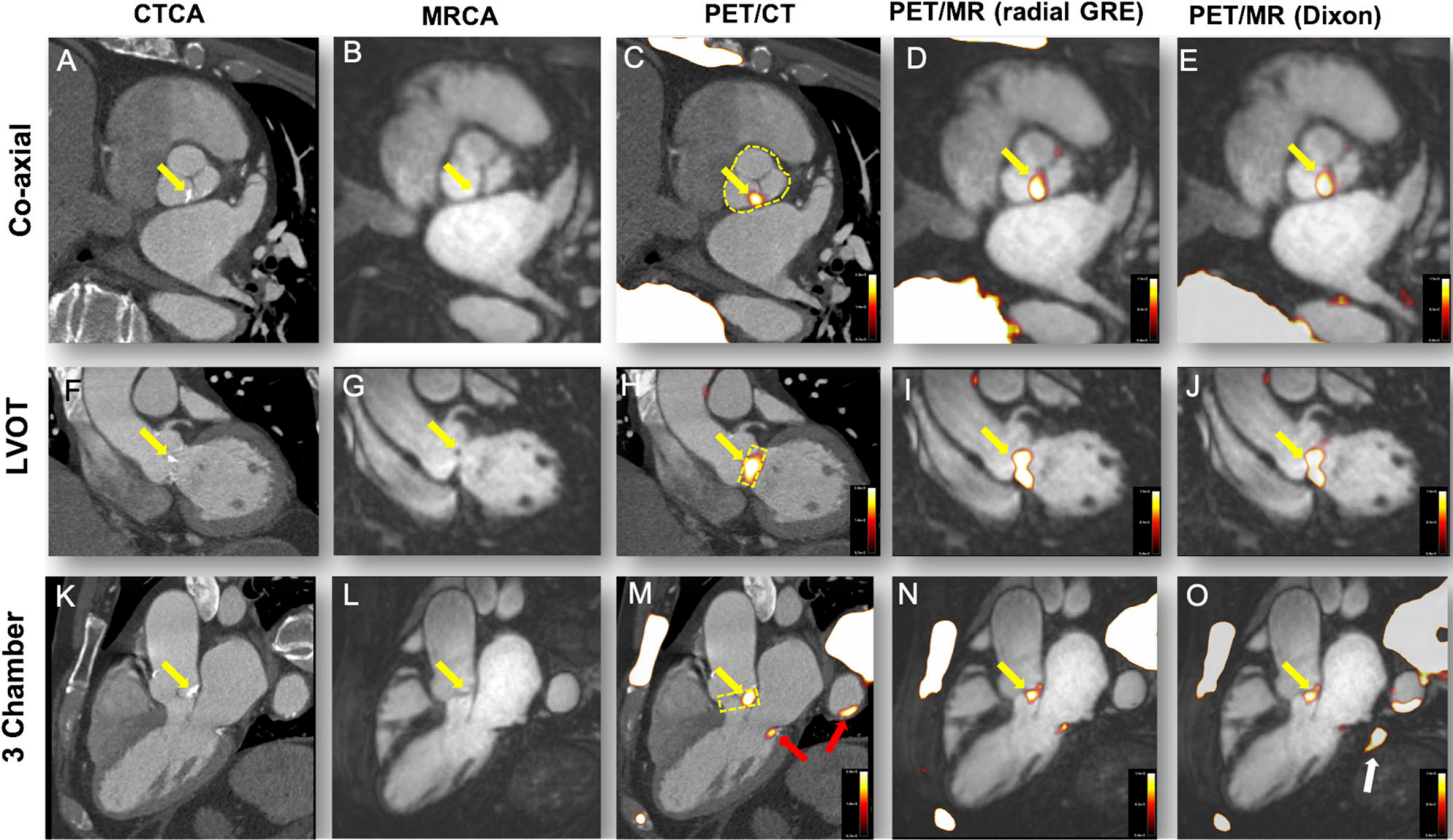
^18^F-Fluoride uptake in a patient with moderate aortic stenosis. The columns represent the imaging modality and rows the corresponding view. (**A**, **F**, **G**) Calcification of the aortic valve (non-coronary cusp predominantly, yellow arrows). (**B**, **G**, **L**) The coronary magnetic resonance angiogram in the same views. Calcification cannot be appreciated on MR but the raphe between the non-coronary cusp and left coronary cusp appears thickened (**B**). PET/CT shows uptake overlaying these areas of calcification (**C**, **H**, **M**). Note the uptake also over the calcified mitral annulus (**M**, red arrow) and arterial wall of the descending aorta (**M**, red arrow). Radial GRE-fused PET/MR shows ^18^F-fluoride uptake in the same areas as the PET/CT (**D**, **I**, **N**). (**E**, **J, O)** The corresponding views in the Dixon PET/MR attenuation correction map. Again ^18^F-Fluoride follows a similar pattern to PET/CT but note the image artifact in (**O**) (white arrow)

Intense aortic valve ^18^F-fluoride uptake was observed in all seven patients with aortic stenosis on both PET/CT and PET/MR (Figure 2). Moreover, the pattern of tracer uptake with the valve was similar across the two modalities, localizing predominantly to the tips of the valve leaflets and the commissures (Figure 2).

**Figure 3:**
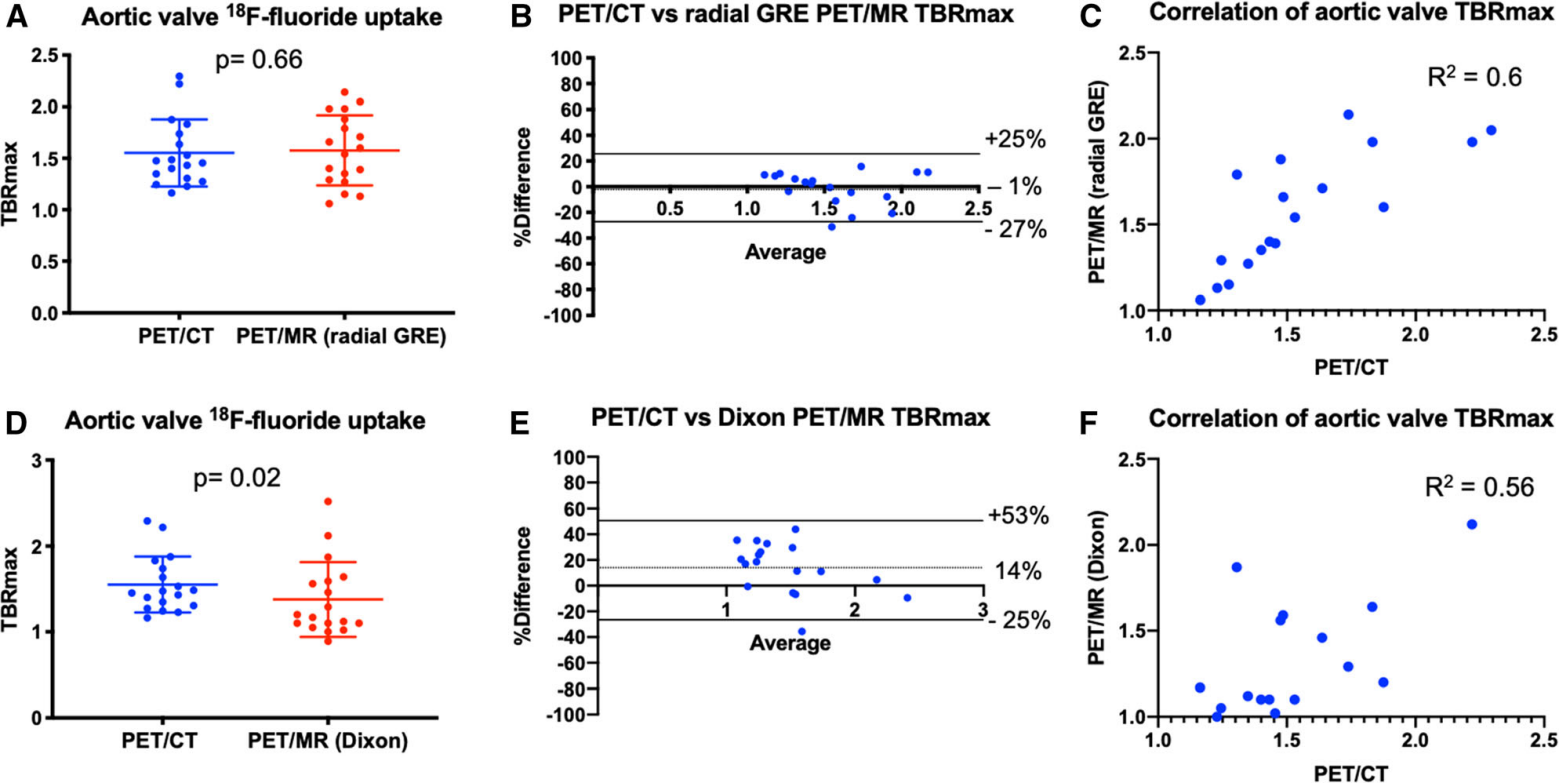
Comparison of PET/CT vs both PET/MR attenuation correction techniques when sampling the aortic valve. (**A**) A direct comparison of mean TBR_MAX_ of ^18^F-fluoride uptake on the aortic valve on PET/CT and radial GRE PET/MR (mean with standard deviation). (**B**) The Bland–Altman comparison of ^18^F-fluoride uptake in the aortic valve between PET/CT and radial GRE PET/MR. (**C**) The correlation and *R*^2^ value between PET/CT and radial GRE PET/MR. (**D**-**F**) The respective comparison between PET/CT and Dixon PET/MR. Note the significant difference in mean TBR_MAX_ (**D**), wider limits of agreement on the Bland–Altman plot (**E**), and lower *R*^2^ value on the correlation plot (**F**)

Aortic valve TBR_MAX_ values were higher in patients with aortic stenosis than those without: 44% higher using PET/CT, 30% using radial GRE PET/MR and 37% using Dixon PET/MR (Suppl. Figure 3). Across the cohort as a whole, aortic valve SUV_MAX_ values were higher on PET/CT than PET/MR irrespective of the attenuation correction approach (Table 2). Similarly, right atrial blood pool SUV values were higher on PET/CT than both PET/MR attenuation correction techniques even after correcting for differences in injection-to-scan time (Table 2). These two effects canceled each other out so TBR values were similar on PET/MR compared to PET/CT (Figure 3A, B and Table 2).

**Table 2:** Comparison of aortic valve standardized uptake values and tissue-to-background values between PET/CT and both PET/MR attenuation correction maps in all patients

	PET/CT	PET/MR (radial GRE)	Agreement PET/CT vs PET/MR (radial GRE)	PET/MR (Dixon)	Agreement PET/CT vs PET/MR (Dixon)
Aortic valve SUV_MAX_ (*n* = 18)	1.76 ± 0.56	1.23 ± 0.38	*P* = < 0.01	1.22 ± 0.43	*P* = < 0.01
			95% LoA = − 14% to 84%		95% LoA = − 3% to 77%
			Bias = 35%		Bias = 37%
Aortic valve SUV_MEAN_	1.35 ± 0.34	0.89 ± 0.26	*P* = < 0.01	0.85 ± 0.26	*P* = < 0.01
			95% LoA = − 15% to 97%		95% LoA = 10% to 82%
			Bias = 42%		Bias = 47%
TC Aortic valve TBR_MAX_	1.55 ± 0.33	1.58 ± 0.34	*P* = > 0.99	1.38 ± 0.44	*P* = 0.06
			95% LoA = − 28% to 25%		95% LoA = − 25% to 53%
			Bias = − 1%		Bias = 13%
TC Aortic valve TBR_MEAN_	1.21 ± 0.18	1.12 ± 0.15	*P* = 0.08	0.90 ± 0.24	*P* = < 0.01
			95% LoA = − 19% to 34%		95% LoA = − 9% to 59%
			Bias = 8%		Bias = 25%
TC Right atrium SUV_MEAN_	1.14 ± 0.24	0.80 ± 0.23	*P* = < 0.01	0.90 ± 0.25	*P* = < 0.01
			95% LoA = − 24% to 98%		95% LoA = − 31% to 79%
			Bias = 36%		Bias = 24%

*PET/CT*, Positron Emission Tomography/Computerized Tomography; *PET/MR*, Positron Emission Tomography/Magnetic Resonance; *SUV*, standardized uptake value; *TBR*, tissue-to-background ratio; *TC*, time-corrected

On Bland–Altman analysis, there were no fixed or proportional biases in TBR_MAX_ values between radial GRE PET/MR and PET/CT (bias − 1% limits of agreement − 27% to + 25%) (Figure 3B; Table 3). Intra-class correlation coefficient (ICC) was good to excellent at 0.878. Agreement between Dixon PET/MR and PET/CT TBR_MAX_ values remained good but was less strong (14% bias, limits of agreement − 25% to + 53%; ICC = 0.794, Figure 3E; Table 2). Generally, agreement between TBR_MEAN_ values on PET/CT and PET/MR was less good than for TBR_MAX_ (Table 2).

**Table 3:** Comparison of time-corrected coronary standardized uptake values and tissue-to-background values between PET/CT and both PET/MR maps in all patients

	PET/CT (60 minutes)	PET/MR (radial GRE)	Agreement PET/CT vs PET/MR (radial GRE)	PET/MR (Dixon)	Agreement PET/CT vs PET/MR (Dixon)
Non-stented coronary plaque SUV_MAX_ (*n* = 28)	1.08 ± 0.29	0.85 ± 0.28	*P* = < 0.01	0.86 ± 0.24	*P* = < 0.01
			95% LoA = − 48% to 97%		95% LoA = − 32% to 77%
			Bias = 25%		Bias = 32%
Stented coronary SUV_MAX_ (*n* = 18)	1.34 ± 0.33	0.47 ± 0.25	*P* = < 0.01	0.77 ± 0.18	*P* = < 0.01
			95% LoA = 3% to 191%		95% LoA = − 9% to 114%
			Bias = %		Bias = 52%
TC non-stented coronary plaque TBR_MAX_ (*n* = 28)	1.09 ± 0.19	1.24 ± 0.27	*P* = 0.03	1.09 ± 0.26	*P* = > 0.99
			95% LoA = − 54% to 31%		95% LoA = − 41% to 42%
			Bias = − 11%		Bias = 1%
TC stented coronary TBR_MAX_ (*n* = 18)	1.28 ± 0.34	0.58 ± 0.31	*P* = < 0.01	0.89 ± 0.24	*P* = < 0.01
			95% LoA = − 14% to 172%		95% LoA = − 5% to 75%
			Bias = 79%		Bias = 359%

*PET/CT*, Positron Emission Tomography/Computerized Tomography; *PET/MR*, Positron Emission Tomography/Magnetic Resonance; *SUV*, standardized uptake value; *TBR*, tissue-to-background ratio; *TC*, time-corrected

### Coronary ^18^F-Fluoride Uptake

Visual agreement in determining coronary ^18^F-fluoride uptake on PET/CT and PET/MR was generally good in non-stented regions. Across the total population, a total of 28 (1.56 plaques/patient) non-stented coronary plaques demonstrated increased ^18^F-fluoride uptake on PET/CT. PET/MR identified excellent agreement with increased uptake in 28 (97%) of these lesions irrespective of the method of attenuation correction (*κ* 0.93, CI 0.837 to 1.000). Four plaques demonstrated increased ^18^F-fluoride uptake on the PET/MR scans but not on PET/CT.

**Figure 4:**
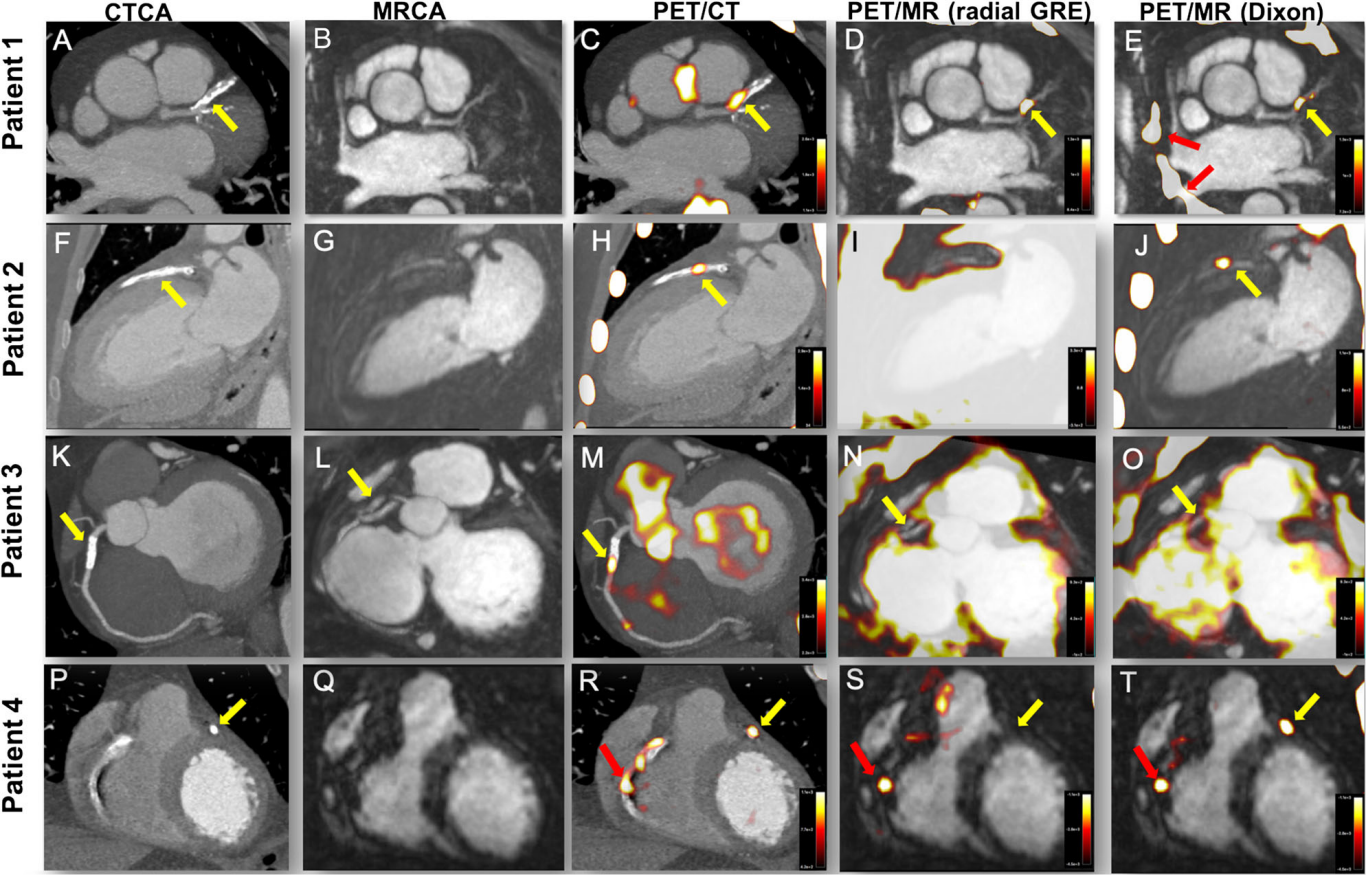
Influence of stents on coronary artery ^18^F-fluoride uptake. Each row represents a patient and the columns the imaging modality. Patient 1: Axial view of complex calcified plaque in the proximal left anterior descending artery (pLAD) (yellow arrow, **A**) on CCTA. The corresponding CMRA can be appreciated in (**B**). PET/CT (**C**) shows focal uptake overlying the complex plaque in the pLAD (yellow arrow) and uptake in the medial wall of the aorta. Both the radial GRE (**D**) and Dixon PET/MR (**E**) demonstrate focal uptake within the LAD plaque (yellow arrows) as the PET/CT. However, note the absence of uptake within the aorta demonstrating the utility of later imaging in improving signal-to-noise ratios. Also note airway artifact behind the behind the left atrium and superior vena cava (red arrows) with the Dixon PET/MR (**E**). Patient 2: Anterior myocardial infarction with primary PCI to the LAD. (**F**) The 2-chamber view of the metallic stent on the CCTA (yellow arrow). (**G**) The corresponding CMRA. Focal ^18^F-fluoride uptake can be appreciated on the PET/CT within the body of the stent (**H**, yellow arrow). (**I**) The radial GRE PET/MR affected by severe PET dropout over the whole stent despite marked amplification of the blood pool. Employing the Dixon AC map sees the culprit artery signal within the LAD stent return (**J**, yellow arrow). Patient 3: Modified short-axis CCTA with stent in the proximal RCA (yellow arrow). (**L**) The corresponding CMRA image of the stent (yellow arrow). (**M**) Focal ^18^F-fluoride uptake over the body of the stent (yellow arrow) on PET/CT. Radial GRE PET/MR shows PET dropout over the body of the stent (**N**, yellow arrow). Similarly, Dixon PET/MR is affected by the same artifact precluding assessment of PET activity within the stent (**O**, yellow arrow). Patient 4: Short-axis CCTA (**P**) shows a diseased right coronary artery (RCA) and a stent placed in the pLAD (yellow arrow) after primary PCI. Corresponding CMRA can be appreciated in (**Q**). Uptake within the proximal to mid RCA (red arrow) and culprit LAD (yellow arrow) can be appreciated on the PET/CT (**R**). PET/MR also shows focal ^18^F-fluoride uptake in the mid RCA with radial GRE PET/MR (S, red arrow) but note the absence of LAD uptake in the region of the stent (yellow arrow). On Dixon PET/MR (**T**), the LAD uptake is visible (yellow arrow) alongside focal RCA uptake (red arrow)

**Figure 5:**
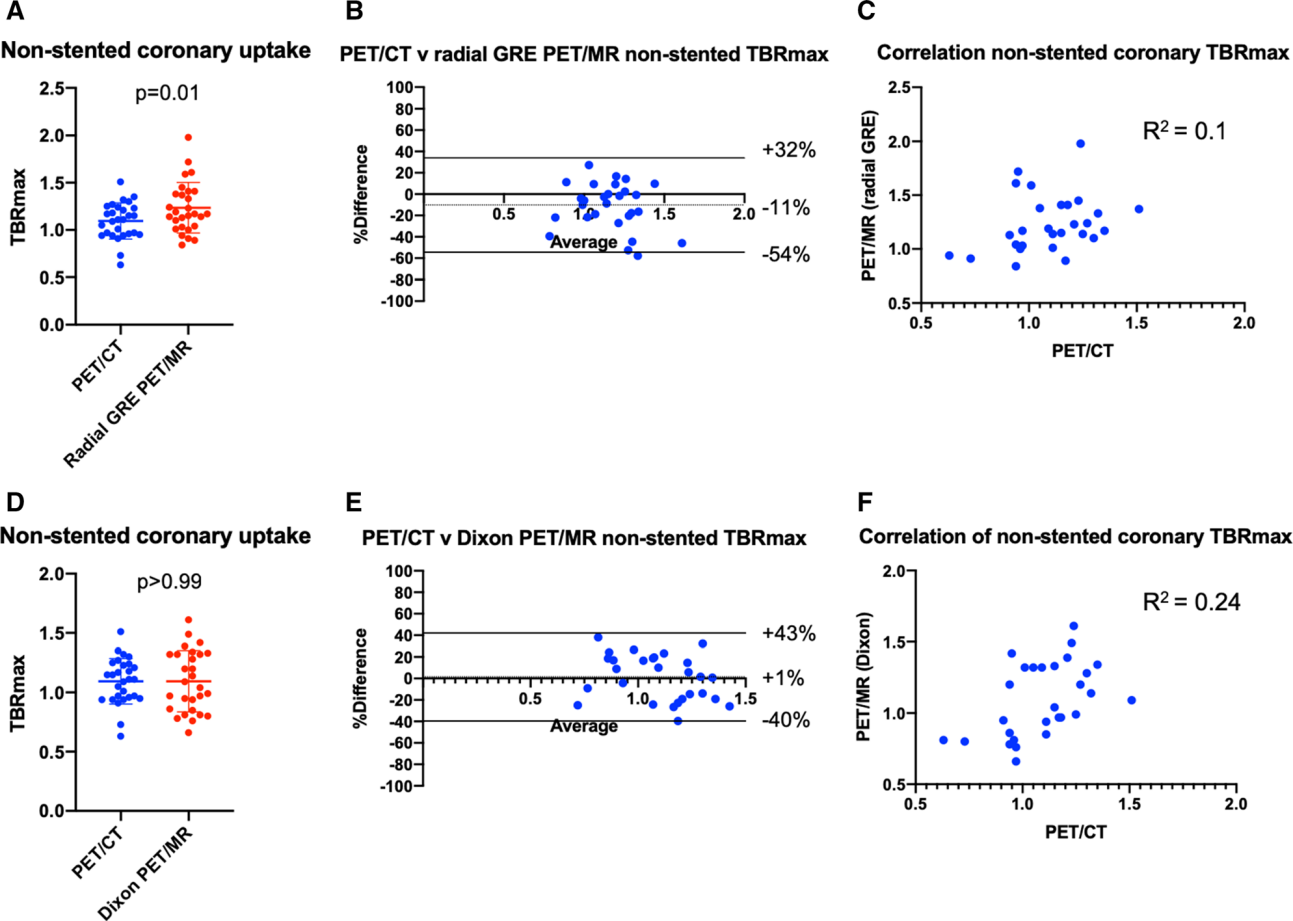
Comparison of PET/CT vs both PET/MR attenuation correction techniques when sampling non-stented coronary uptake. (**A**) A direct comparison of mean TBR_MAX_ of ^18^F-fluoride uptake in non-stented coronaries on PET/CT and radial GRE PET/MR (mean with standard deviation). Note how uptake is significantly higher with radial GRE PET/MR when compared to PET/CT. (**B**) The Bland–Altman comparison (with 95% limits of agreement) of coronary ^18^F-fluoride uptake between PET/CT and radial GRE PET/MR. (**C**) The correlation and *R*^2^ value between PET/CT and radial GRE PET/MR in non-stented coronaries. (**D**-**F**) The respective comparison between PET/CT and Dixon PET/MR. Note the lower mean TBR_MAX_ for Dixon PET/MR when compared to radial GRE (**D**). Dixon PET/MR had less bias on the Bland–Altman plot (**B**) and a higher *R*^2^ value on the correlation plot (**F**)

In the native coronary arteries, SUV_MAX_ values were lower on PET/MR (using both attenuation correction techniques) than on PET/CT consistent with findings in the valve. TBR_MAX_ values were comparable, although slightly higher using GRE PET/MR than PET/CT (bias − 11%, limits of agreement − 54% to 32%; Table 3; Figure 4B, 5).

Coronary stents caused severe artifact on both the radial GRE and Dixon MR attenuation correction maps, resulting in marked dropout of the PET signal and precluding accurate analysis. Agreement between PET/CT and PET/MR in determining ^18^F-fluoride uptake within coronary stents was therefore poor (Table 3). Increased ^18^F-fluoride uptake was observed on PET/CT in 18 coronary stent segments. On radial GRE PET/MR, ^18^F-fluoride uptake in the body of the stent was obscured by artifact in all 18 stents, although increased uptake could be appreciated either at the proximal or distal end of the stent in 6 cases. Artifact was also observed with Dixon PET/MR, although it was less pronounced than the radial GRE PET/MR (Figure 4O), with increased ^18^F-fluoride activity observed at the margins of the stent in 7 cases (Suppl. Figure 3). Interestingly this artifact was not present in heavily calcified arteries or valves on either PET/MR AC map. The differences in tissue classification between both PET/MR attenuation maps can be further appreciated in Supplementary Figure 1.

## Discussion

We have compared ^18^F-fluoride PET/MR with PET/CT imaging of the aortic valve and coronary arteries in cohorts of patients with aortic stenosis or coronary heart disease. We have shown that the pattern of ^18^F-fluoride uptake within the aortic valve and in non-stented coronaries is similar on both scans, although coronary PET/MR is limited by artifact at the site of intra-coronary stent implantation. Quantitatively, across both valve and non-stented coronary arteries SUV_MAX_ uptake values are greater on PET/CT than PET/MR. This difference is however corrected for in the calculation of TBR_MAX_ values thereby supporting the future use of ^18^F-fluoride PET/MR in the investigation of aortic stenosis and coronary atherosclerosis.

In this study, we have explored two different MR methods of attenuation correction and made comparison with PET/CT as the reference standard arbitrator. As previously reported, we have demonstrated that Dixon is consistently affected by extra-cardiac artifacts in the bronchus and heart–lung and lung–diaphragm interfaces. This problem is resolved with the use of a free-breathing radial GRE attenuation correction sequence which also consistently provided improved agreement with PET/CT-derived TBR and SUV values. We would therefore recommend use of the radial GRE attenuation correction method in cardiovascular PET/MR studies.

^18^F-fluoride PET/CT quantifies calcification activity and predicts disease progression in aortic stenosis and other valve conditions.^13^,^15^–^17^ It is currently being explored as an efficacy endpoint in studies of novel therapies for aortic stenosis including the SALTIRE 2 (NCT02132026) randomized controlled trial. If tracer uptake could also be quantified with PET/MR then this modality would hold many advantages, not least the greatly reduced radiation exposure: a particularly important consideration given the need in such trials for serial scans. ^18^F-Fluoride PET/MR is being explored as an efficacy endpoint for these exact reasons in the BASIK 2 trial (Bicuspid Aortic Valve Stenosis and the Effect of Vitamin K2 on Calcification Using ^18^F -Sodium Fluoride Positron Emission Tomography/Magnetic Resonance, NCT02917525).^18^ Establishing whether PET/MR provides similar results to PET/CT is therefore important.

In this study, we have demonstrated that the pattern of valvular ^18^F-fluoride uptake on PET/MR was reassuringly similar to that observed on PET/CT, with high intensity uptake localizing to the valve in all 7 patients with aortic stenosis (Figure 1 and Suppl. Figure 3).^10^,^13^,^14^ Interestingly, this uptake appeared to localize to the leaflet edges and the commissures: the sites of maximal mechanical stress within the valve.

Imaging of the coronary arteries with PET/MR does present additional challenges driven by the small size and complex motion of the coronary arteries. We have demonstrated successful use of Gadobutrol contrast for CMRA (when administered as a slow infusion). This CMRA technique allowed clear appreciation of the proximal two-thirds of each epicardial artery (Figure 3B) facilitating localisation of ^18^F-uptake to the coronary arteries.

As with previous studies,^15^,^16^ uptake of ^18^F-fluoride in culprit coronary plaques post myocardial infarction was common. Moreover, the pattern of ^18^F-fluoride uptake in native coronary arteries on PET/CT and PET/MR was very similar, with 28 of the 29 plaques with increased tracer activity on PET/CT also identified on PET/MR. Radial GRE PET/MR TBR_MAX_ values in these areas were again comparable to PET/CT although limits of agreement were slightly wider than for the valve. This may reflect partial volume effects or subtle differences in PET/MR tissue classification at the cardiac-lung boundary of the AC maps. Our data are also consistent with the original findings by Robson et al^3^ as we observed higher coronary TBR_MAX_ values on radial GRE PET/MR compared to Dixon although quantitative agreement with PET/CT was less good (Figure 5B).

Unfortunately, coronary PET/MR is greatly hampered by artifact at the site of intra-coronary stent implantation. This is due to magnetic field inhomogeneities and subsequent MR signal loss affecting attenuation correction of the PET data (Suppl. Figure 1). It was particularly prevalent with radial GRE PET/MR, where substantial ‘halo’ like PET dropout occurred at the site of all stents imaged (Figure 4I+N and Suppl. Figure 3B). Although less dramatic with the Dixon PET/MR scans, the degree of artifact was such that reliable interpretation of PET signal remained impossible (Suppl. Figure 3C). Attempts to overcome this limitation using novel MR gap-filling algorithms have shown early promise.^19^ Interestingly, areas of dense coronary and valvular calcification were not affected by PET dropout.

Discrepancies were observed when comparing SUV values in the valve and coronary arteries measured by PET/CT and PET/MR approaches (Tables 2, 3). SUV values were consistently higher on PET/CT than PET/MR. This may be explained by basic differences in the cardiac PET/CT and PET/MR imaging protocols. First, approaches to attenuation correction are fundamentally different between PET/CT and PET/MR. Second PET/CT requires the patients’ arms to be held above their head. This is designed to not only reduce radiation dose but also to minimize photon attenuation under the shoulders and reduce beam hardening artifacts.^20^ The bore of the PET/MR scanner is much smaller, and the arms are therefore held by the patients’ side. This undoubtedly causes minor shifts in cardiac position affecting PET photon lines of response, attenuation and subsequent SUV values.^21^,^22^ A paired 18F-fluoride carotid artery study would help quantify the true difference (free from photon attenuation by the arms) between PET/CT and PET/MR. Thirdly, the injection-to-scan interval was longer for PET/MR than PET/CT. Given ^18^F-fluoride SUV values have been shown to remain unaltered between 1 and 3 hours of PET/CT imaging,^14^ inter-modality SUV disparity is therefore most likely attributable to the above technical differences (e.g., MR coils^23^) between PET/CT and PET/MR scanners.

In contrast TBR values were similar on PET/CT and PET/MR. Adjustment for blood pool activity effectively corrected TBR values for the between-scanner differences in PET SUV quantification outlined above. Moreover, we adjusted TBR values for differences in injection-to-scan time. After these corrections, we found no differences in uptake values between PET/CT and radial GRE PET/MR (Figure 3; Table 2) over the aortic valve. In non-stented coronary arteries, TBR values tended to be higher on PET/MR than PET/CT allowing easier scan interpretation and highlighting the advantages of later time point imaging (Table 3; Figure 4).^14^

This study is unique in performing cardiac PET/MR immediately after PET/CT, allowing the fairest comparison between the modalities. Nevertheless, there are a number of limitations. Firstly, there are the unavoidable differences in injection-to-scan time for the PET/CT and PET/MR scans. Whilst we have employed measures to correct for this impact an alternative approach would have been to scan subjects with PET/CT and PET/MR on two separate occasions. However, this approach would cause other problems that are not as easily fixed as differences in injection-to-scan time. In particular it would involve differences in the injected dose of tracer, small differences in scan to injection time, differences related to variation in the biodistribution of tracer on different days and most importantly increased exposure of patients to ionizing radiation. Second, this was a small pilot investigational study and we would welcome confirmation of our findings in a larger study, with further investigation of quantitative agreement between PET/CT and PET/MR in particular. Third, whilst the observed agreements between PET/CT and PET/MR are encouraging, there are several technical strategies not employed here that could be used to enhance this agreement further. In order to make a fair comparison between modalities, PET data for both PET/CT and PET/MR were not ECG gated nor was time of flight employed. This rendered PET uptake susceptible to motion artifact. Correcting for motion has consistently been shown to improve coronary discrimination and TBR values.^24^–^27^ Fourth, whilst the radial GRE attenuation correction technique reduced PET artifact and generally improved TBR values, it only includes two tissue classes (background air and soft tissue). Increasing the amount of tissue classes is likely to improve uptake discrimination and tracer quantification. Applying the CT attenuation correction map to the PET/MR data is one potential method to assess the precision of both PET/MR attenuation correction maps with the ground truth but arm position remains a limiting factor. Finally, in the era of machine learning, it may be possible to create a pseudoCT attenuation correction map from the MR data and if accurate, would advance the use of the PET/MR as a valid alternative to PET/CT.^28^

In conclusion, valvular and coronary ^18^F-fluoride activity on PET/MR closely matched that observed on PET/CT. Although SUV values differ between PET/CT and PET/MR, the use of TBR values effectively corrects for inter-scanner differences. These data help validate cardiovascular PET/MR imaging, and supports its future investigation of cardiovascular disease, in particular given its advantages in enhanced soft tissue characterisation and reduced radiation exposure.

## New Knowledge Gained

- The pattern of ^18^F-fluoride uptake within the aortic valve and in non-stented coronaries is similar on PET/CT and PET/MR; however, coronary PET/MR is limited by magnetic inhomogeneities at the site of stent implantation.
- Across both valve and non-stented coronary arteries SUV_MAX_ uptake values are greater on PET/CT than PET/MR. This difference is however corrected for in the calculation of TBR_MAX_ values.
- The Dixon method of PET/MR attenuation correction is consistently affected by extra-cardiac artifacts. This problem is largely resolved with the use of a free-breathing radial GRE attenuation correction sequence.

## Electronic supplementary material

Below is the link to the electronic supplementary material.

Supplementary material 1 (PNG 1713 kb)

Supplementary material 2 (PNG 301 kb)

Supplementary material 3 (PNG 1614 kb)

Supplementary material 4 (DOCX 18 kb)

Supplementary material 5 (PPTX 2191 kb)

12350_2019_1962_MOESM6_ESM.mp4

Supplementary material 6 (MP4 6307 kb)

## Abbreviations

PET/CT: Positron Emission Tomography/Computerized Tomography
PET/MR: Positron Emission Tomography/Magnetic Resonance
MRAC: Magnetic Resonance Attenuation Correction
GRE: Gradient Recalled Echo
LGE: Late Gadolinium Enhancement
SUV_MEAN_: Standardized Uptake Value mean
SUV_MAX_: Standardized Uptake value max
TBR_MEAN_: Target-to-Background Ratio mean
TBR_MAX_: Target-to-Background Ratio max
OSEM: Ordered Subsets Expectation Maximization
