# Supplementary figures and images for: Cardiovascular 18F-fluoride positron emission tomography-magnetic resonance imaging: A comparison study
Source: J Nucl Cardiol. 2019 Dec 2;28(5):1–12. doi: 10.1007/s12350-019-01962-y (PMC8616877; doi:10.1007/s12350-019-01962-y)

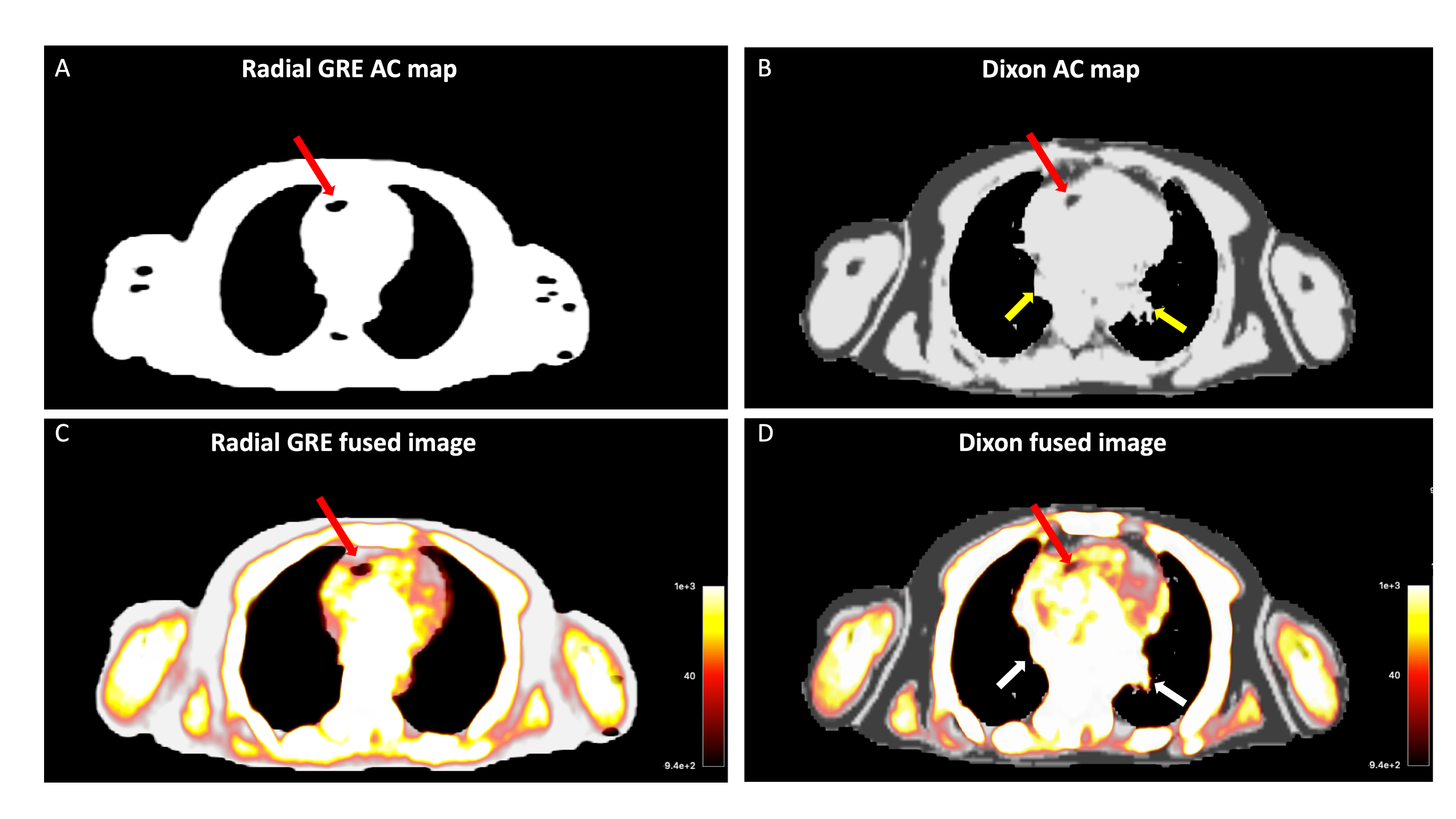

Supplement: Supplementary file 1 — Supplementary material 1 (PNG 1713 kb) [file 12350_2019_1962_MOESM1_ESM.png]

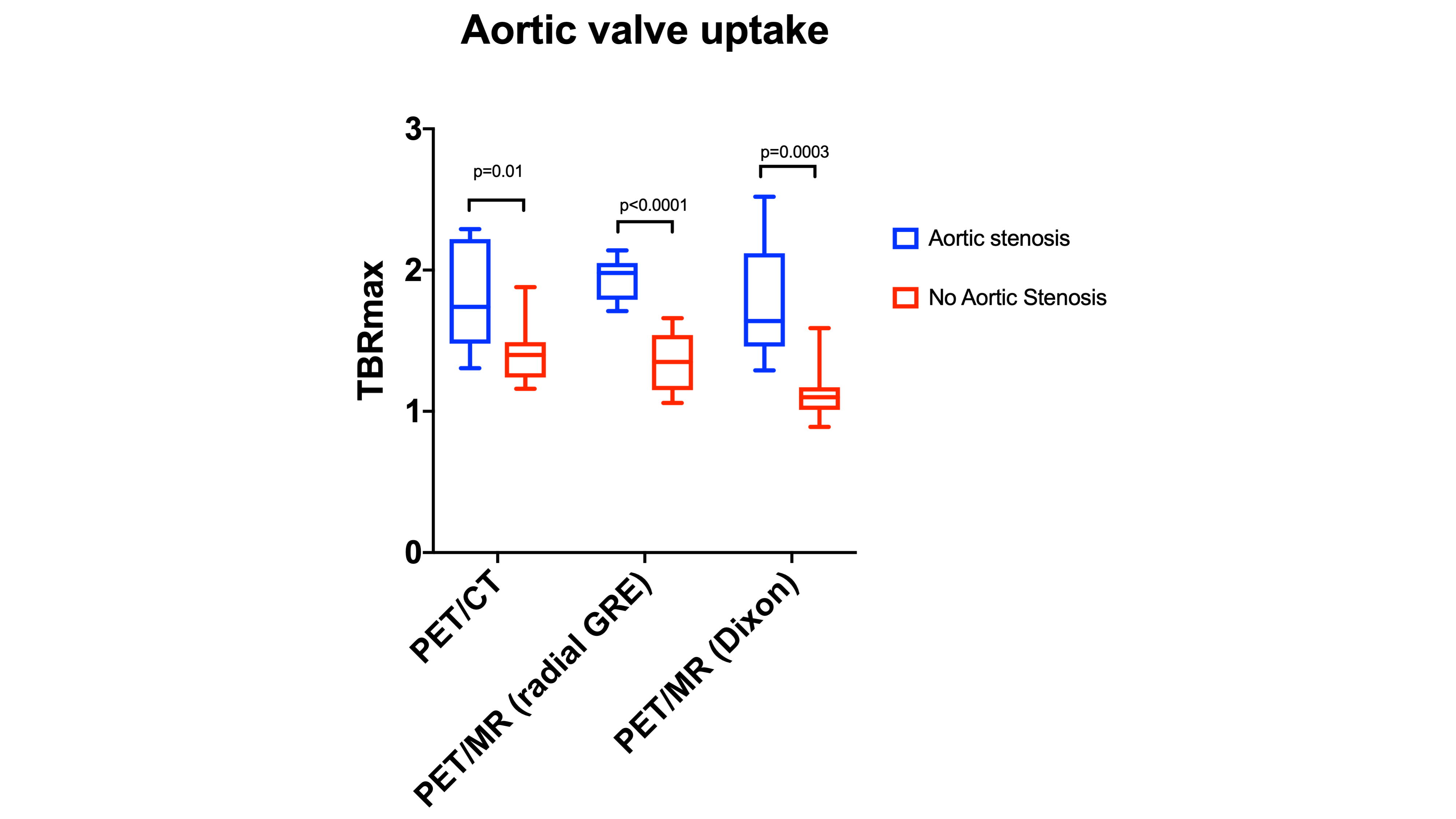

Supplement: Supplementary file 2 — Supplementary material 2 (PNG 301 kb) [file 12350_2019_1962_MOESM2_ESM.png]

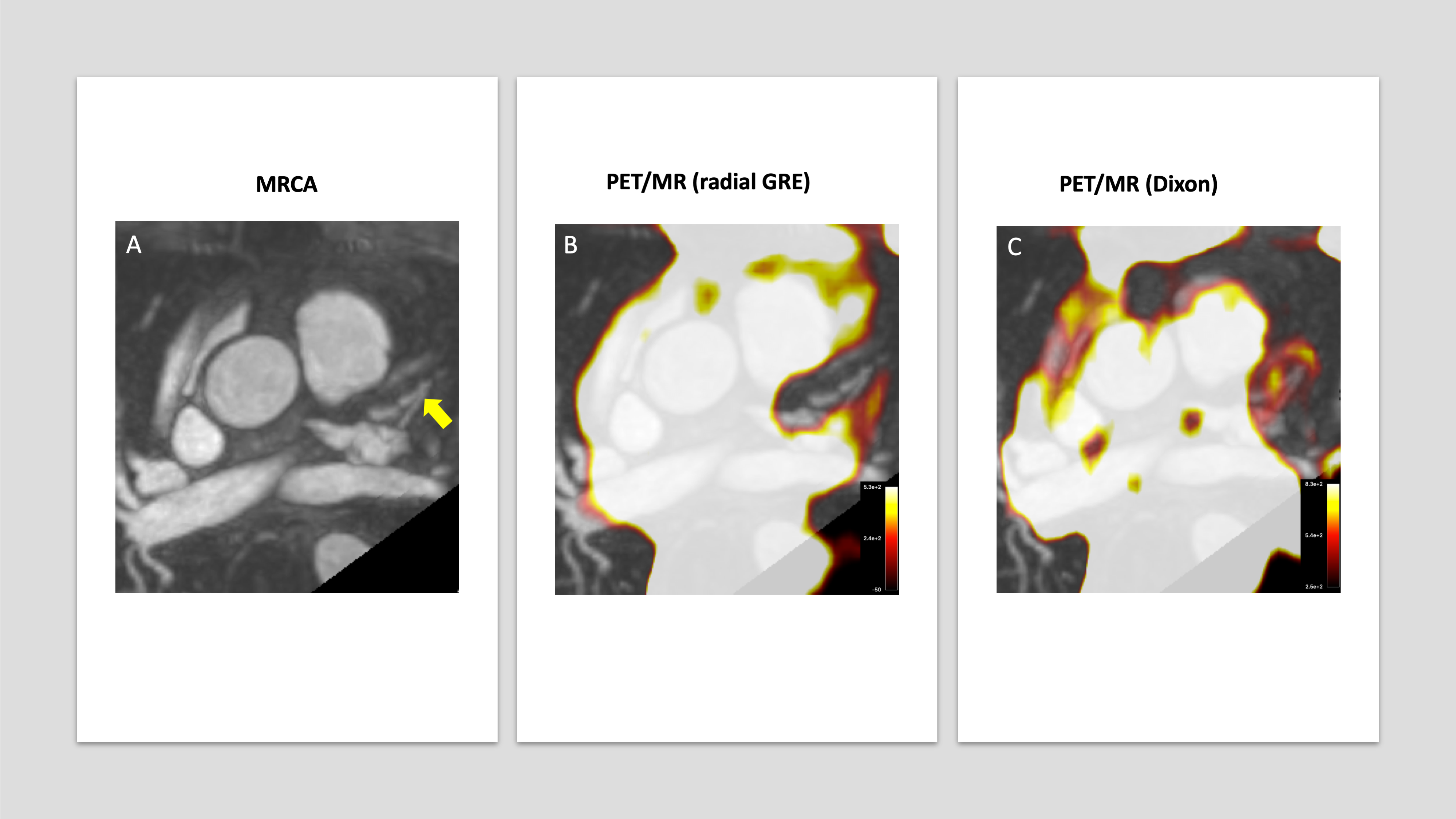

Supplement: Supplementary file 3 — Supplementary material 3 (PNG 1614 kb) [file 12350_2019_1962_MOESM3_ESM.png]
