# Supplementary material for: Cardiovascular 18F-fluoride positron emission tomography-magnetic resonance imaging: A comparison study
Source: J Nucl Cardiol. 2019 Dec 2;28(5):1–12. doi: 10.1007/s12350-019-01962-y (PMC8616877; doi:10.1007/s12350-019-01962-y)
Supplement: Supplementary file 4 — Supplementary material 4 (DOCX 18 kb) [file 12350_2019_1962_MOESM4_ESM.docx]

**PET-CT Valvular, Aortic and Coronary Analysis Protocol**

**Version 1.0, July 2018**

Start FusionQuant software

Load CT coronary angiogram in diastole at 65-70% R-R interval

Load attenuation corrected (x2 zoom) PET data as “overlay”:

- Use summed gate and ‘smooth’ thresholding method.

1. Find aortic valve plane (CT method)

Draw polygon ROI in short axis view

Depth 6mm (similar to CT method) centred on valve

Record

- **AV SUV max**
- **AV SUV mean**

1. On sagittal plane, find the base of right main pulmonary artery where it crosses ascending aorta

Switch to axial view and draw polygon around ascending aorta

Record at least 5 ROI’s of 3mm depth extending cranially

Record

**- Asc Aorta SUVmax**

**- Asc Aorta SUVmean**

1. Reorientate views to show 4Ch, 2Ch and short axis at mid-ventricle level, centre image on LV cavity. Keeping in same 4Ch plane:

Draw spherical ROI in middle of RA cavity (8mm radius, approx. 2cm^3^ volume)

Record

- **RA background SUV mean**

1. Save contours within patient study folder

**PET/MR Valvular, Aortic and Coronary Analysis Protocol**

**Version 1.0, July 2018**

Start FusionQuant software

Load MR coronary angiogram sequence as “background”

Load MRAC-corrected PET data as “overlay”:

- Gate 1: Siemens 3D Dixon VIBE
- Gate 2: NYC radial GRE VIBE

1. Find aortic valve plane (CT method)

Draw polygon ROI in short axis view

Depth 6mm (similar to CT method) centred on valve

Record in both gates:

- **AV SUV max**
- **AV SUV mean**

1. On the axial plane examine for PET uptake within the coronary arteries

increased activity is recorded in spheres of 5mm radius set over hottest area

if;

Uptake localized to a disease coronary artery

Uptake on multiple slices

Uptake on more than 1 reformatted plane

Record in both gates;

**- Coronary SUVmax**

1. Reorientate views to show 4Ch, 2Ch and short axis at mid-ventricle level, centre image on LV cavity. Keeping in same 4Ch plane:

Draw spherical ROI in middle of RA cavity (8mm radius, approx. 2cm^3^ volume)

Record in both gates;

**- RA background SUV mean**

1. Save contours within patient study folder

**Supplemental Figures**

**Supplementary figure 1. Similarities and differences in tissue classification between radial GRE and Dixon attenuation correction maps.** Panel A and B represent an axial view of the respective radial GRE and Dixon MR AC maps for patient 3 from figure 3. Note the signal loss over the area of the stent in the proximal right coronary artery in both AC maps (red arrow, panels A and B) causing corresponding PET signal dropout on the fused images (red arrow, panels C and D). The yellow arrows in panel B represent Dixon tissue misclassification around the heart-lung border (bronchi classed as soft tissue). This is not present in the radial GRE map in panel A. Note how this causes PET artefact within the misclassified areas on the fused image (D, white arrows).

**Supplementary Figure 2. ^18^F-fluoride in uptake in those with and without aortic stenosis.** Comparison of mean TBR_MAX_ values between those with aortic stenosis and those without across PET/CT and both PET/MR AC maps.

**Supplementary Figure 3. Stent related artefact on radial GRE and Dixon PET/MR.** Panel A shows an axial slice of a stent within the proximal LAD artery (yellow arrow). The corresponding radial GRE and Dixon PET/MR can be appreciated in panels B and C respectively. Note the severe PET dropout over the stent with the radial GRE AC in panel B. Whilst dropout still exists with the Dixon method it is less well defined and pronounced (C).
