# Supplementary material for: Cardiovascular 18F-fluoride positron emission tomography-magnetic resonance imaging: A comparison study
Source: J Nucl Cardiol. 2019 Dec 2;28(5):1–12. doi: 10.1007/s12350-019-01962-y (PMC8616877; doi:10.1007/s12350-019-01962-y)
Supplement: Supplementary file 5 — Supplementary material 5 (PPTX 2191 kb) [file 12350_2019_1962_MOESM5_ESM.pptx]

## Slide 1
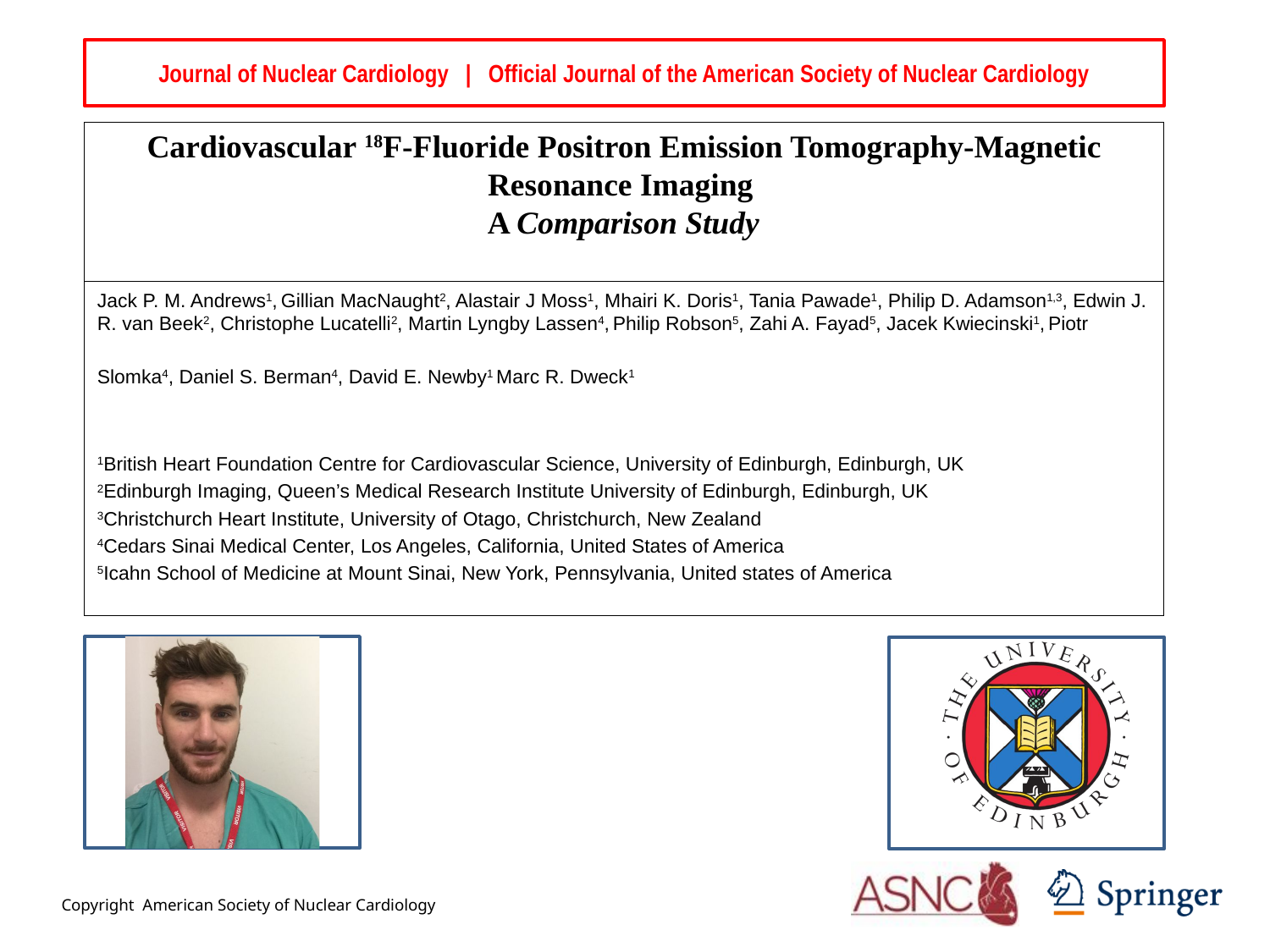

Journal of Nuclear Cardiology | Official Journal of the American Society of Nuclear Cardiology
# Cardiovascular 18F-Fluoride Positron Emission Tomography-Magnetic Resonance Imaging A Comparison Study
Jack P. M. Andrews1, Gillian MacNaught2, Alastair J Moss1, Mhairi K. Doris1, Tania Pawade1, Philip D. Adamson1,3, Edwin J. R. van Beek2, Christophe Lucatelli2, Martin Lyngby Lassen4, Philip Robson5, Zahi A. Fayad5, Jacek Kwiecinski1, Piotr Slomka4, Daniel S. Berman4, David E. Newby1 Marc R. Dweck1
1British Heart Foundation Centre for Cardiovascular Science, University of Edinburgh, Edinburgh, UK
2Edinburgh Imaging, Queen’s Medical Research Institute University of Edinburgh, Edinburgh, UK
3Christchurch Heart Institute, University of Otago, Christchurch, New Zealand
4Cedars Sinai Medical Center, Los Angeles, California, United States of America
5Icahn School of Medicine at Mount Sinai, New York, Pennsylvania, United states of America
Copyright American Society of Nuclear Cardiology

## Slide 2
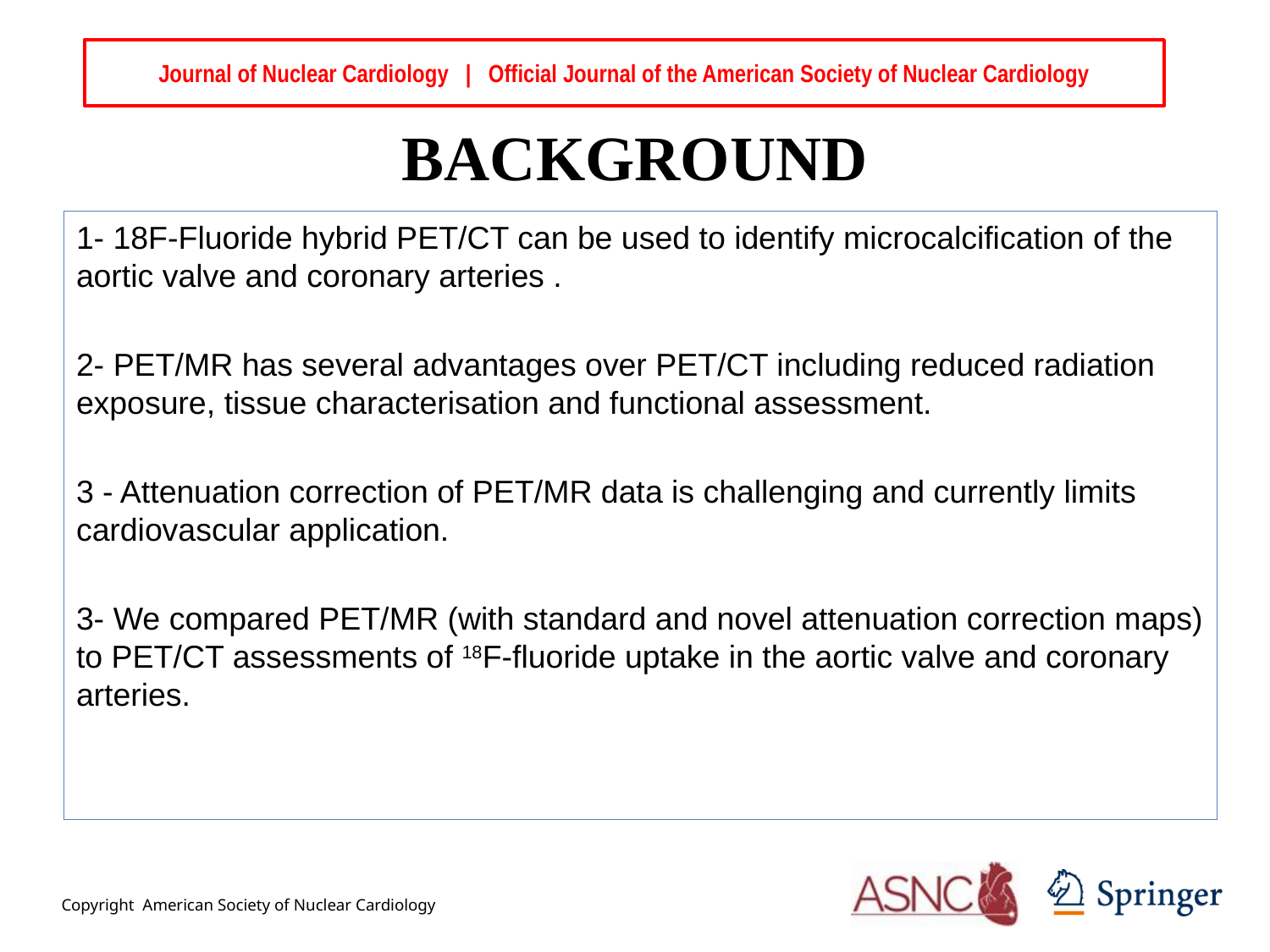

Journal of Nuclear Cardiology | Official Journal of the American Society of Nuclear Cardiology
# BACKGROUND
1- 18F-Fluoride hybrid PET/CT can be used to identify microcalcification of the aortic valve and coronary arteries .
2- PET/MR has several advantages over PET/CT including reduced radiation exposure, tissue characterisation and functional assessment.
3 - Attenuation correction of PET/MR data is challenging and currently limits cardiovascular application.
3- We compared PET/MR (with standard and novel attenuation correction maps) to PET/CT assessments of 18F-fluoride uptake in the aortic valve and coronary arteries.
Copyright American Society of Nuclear Cardiology

## Slide 3
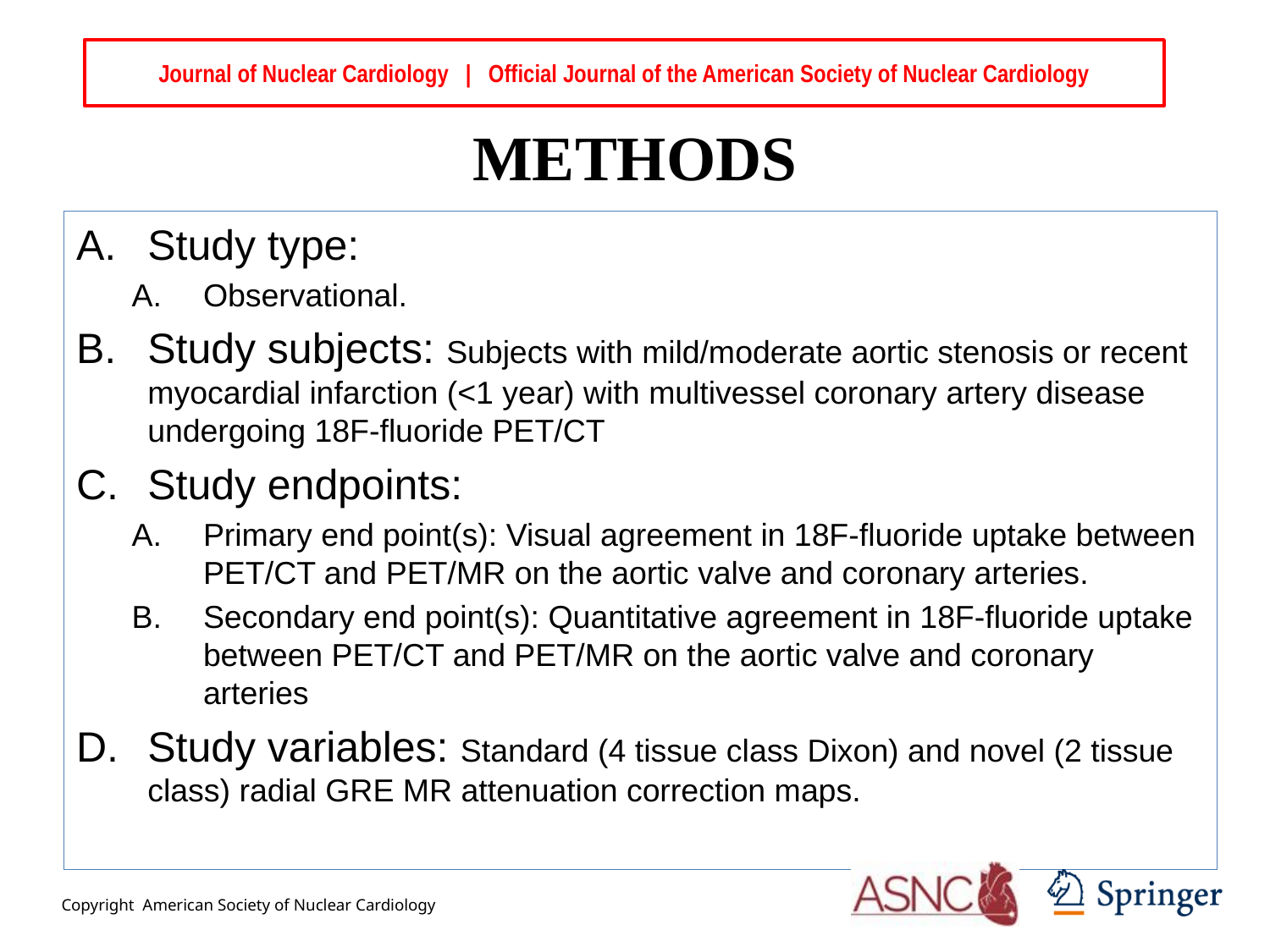

Journal of Nuclear Cardiology | Official Journal of the American Society of Nuclear Cardiology
# METHODS
Study type:
Observational.
Study subjects: Subjects with mild/moderate aortic stenosis or recent myocardial infarction (<1 year) with multivessel coronary artery disease undergoing 18F-fluoride PET/CT
Study endpoints:
Primary end point(s): Visual agreement in 18F-fluoride uptake between PET/CT and PET/MR on the aortic valve and coronary arteries.
Secondary end point(s): Quantitative agreement in 18F-fluoride uptake between PET/CT and PET/MR on the aortic valve and coronary arteries
Study variables: Standard (4 tissue class Dixon) and novel (2 tissue class) radial GRE MR attenuation correction maps.
Copyright American Society of Nuclear Cardiology

## Slide 4
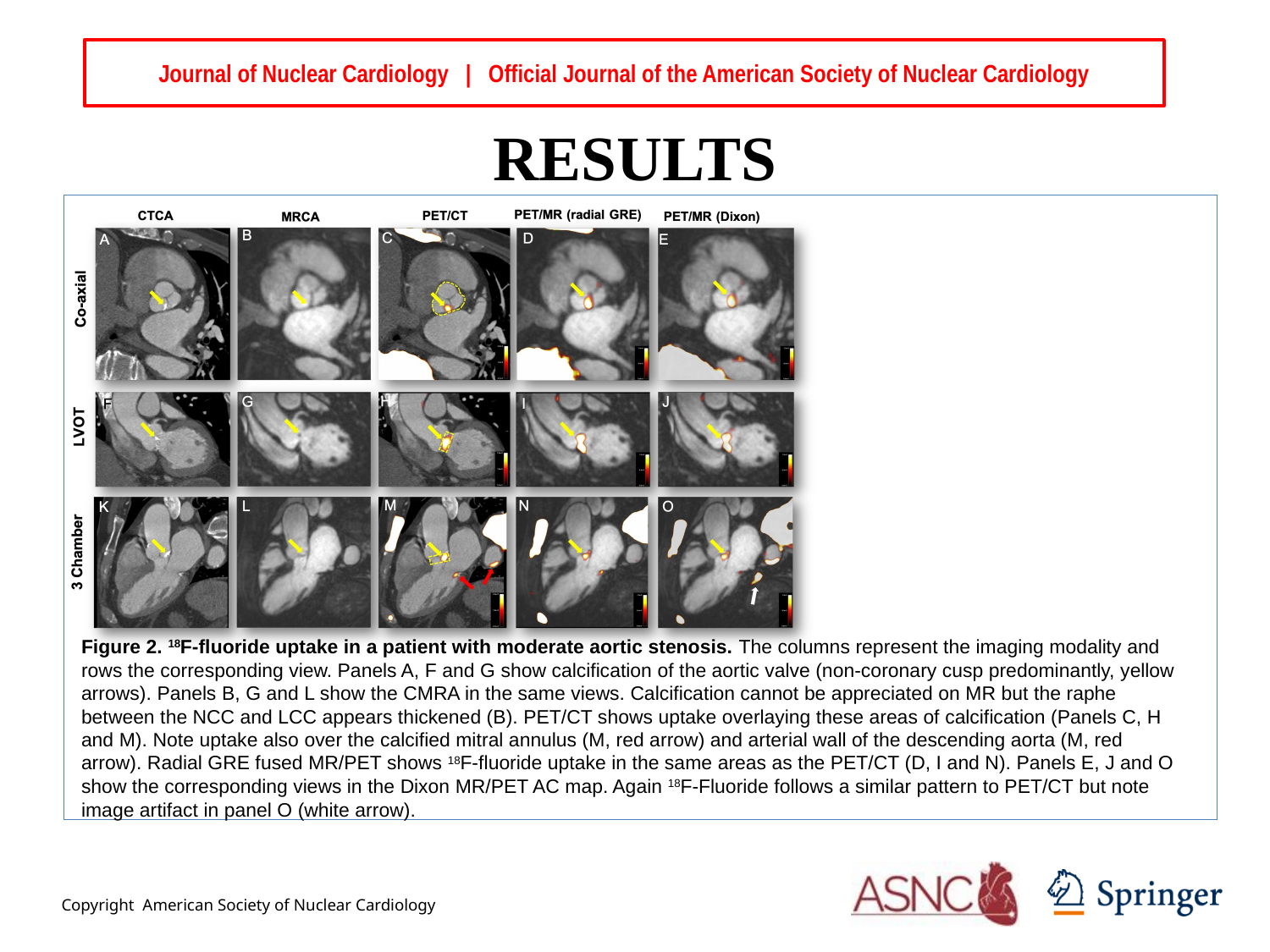

Journal of Nuclear Cardiology | Official Journal of the American Society of Nuclear Cardiology
# RESULTS
Figure 2. 18F-fluoride uptake in a patient with moderate aortic stenosis. The columns represent the imaging modality and rows the corresponding view. Panels A, F and G show calcification of the aortic valve (non-coronary cusp predominantly, yellow arrows). Panels B, G and L show the CMRA in the same views. Calcification cannot be appreciated on MR but the raphe between the NCC and LCC appears thickened (B). PET/CT shows uptake overlaying these areas of calcification (Panels C, H and M). Note uptake also over the calcified mitral annulus (M, red arrow) and arterial wall of the descending aorta (M, red arrow). Radial GRE fused MR/PET shows 18F-fluoride uptake in the same areas as the PET/CT (D, I and N). Panels E, J and O show the corresponding views in the Dixon MR/PET AC map. Again 18F-Fluoride follows a similar pattern to PET/CT but note image artifact in panel O (white arrow).
Copyright American Society of Nuclear Cardiology

## Slide 5
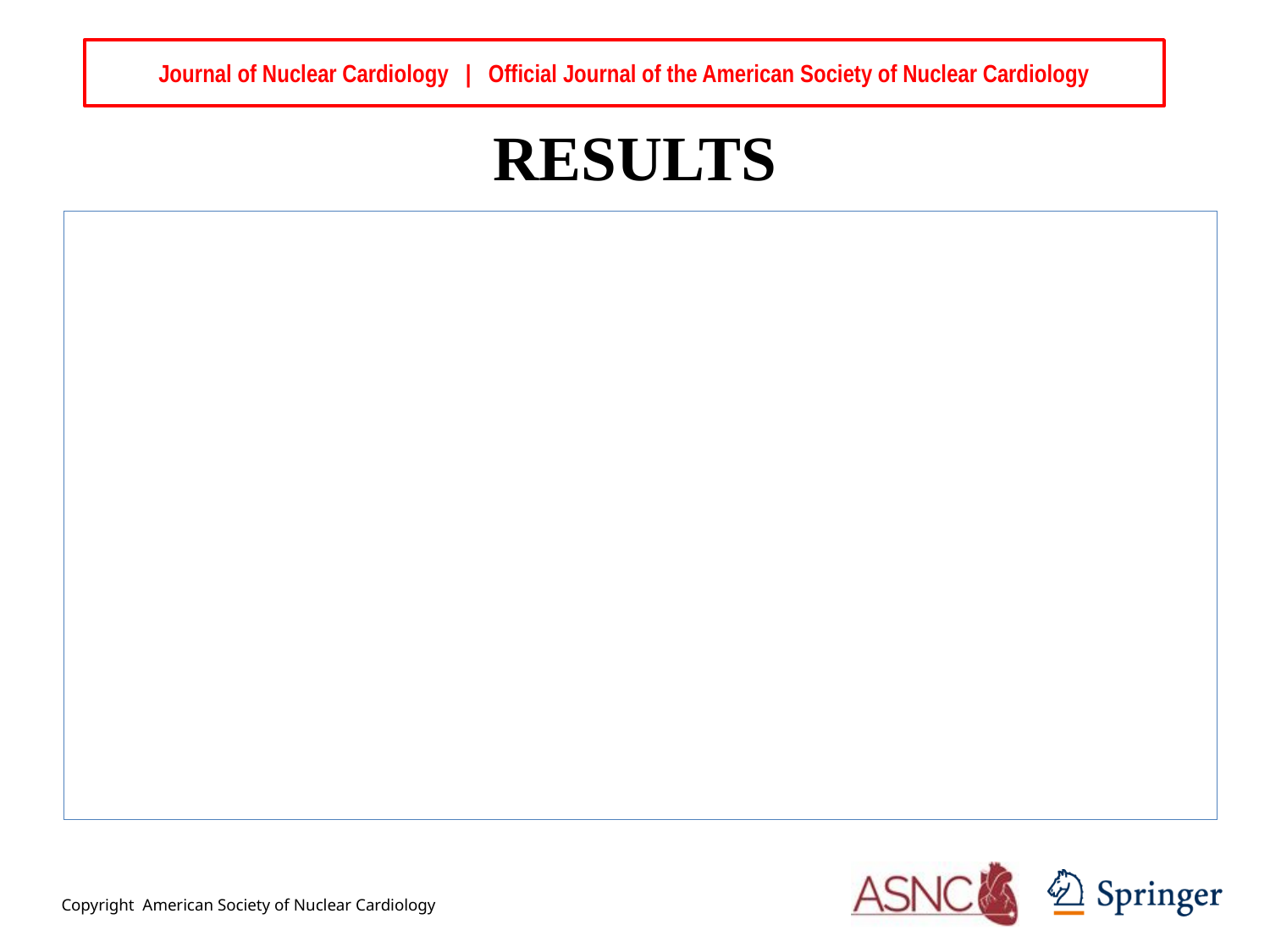

Journal of Nuclear Cardiology | Official Journal of the American Society of Nuclear Cardiology
# RESULTS
Copyright American Society of Nuclear Cardiology

## Slide 6
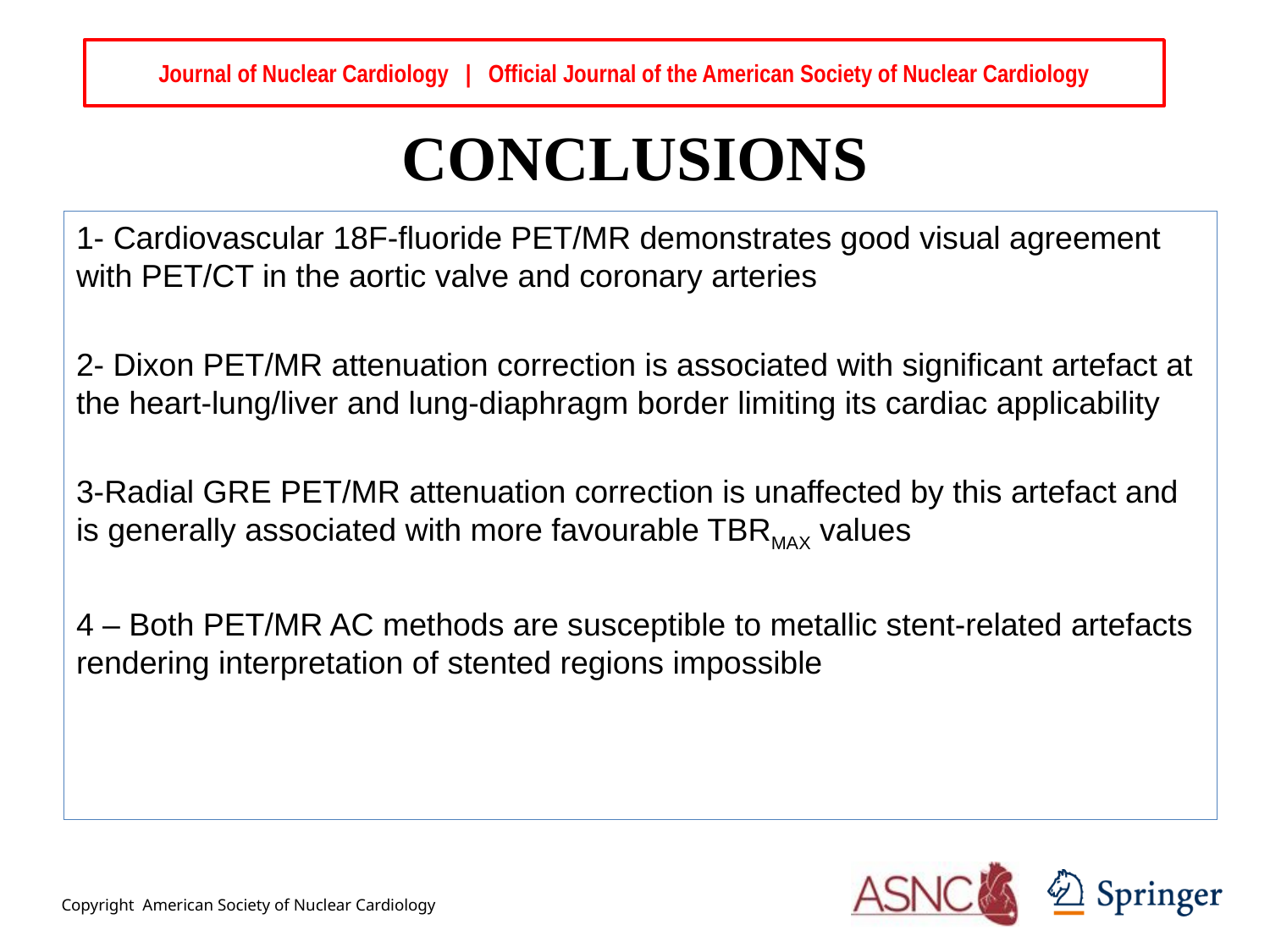

Journal of Nuclear Cardiology | Official Journal of the American Society of Nuclear Cardiology
# CONCLUSIONS
1- Cardiovascular 18F-fluoride PET/MR demonstrates good visual agreement with PET/CT in the aortic valve and coronary arteries
2- Dixon PET/MR attenuation correction is associated with significant artefact at the heart-lung/liver and lung-diaphragm border limiting its cardiac applicability
3-Radial GRE PET/MR attenuation correction is unaffected by this artefact and is generally associated with more favourable TBRMAX values
4 – Both PET/MR AC methods are susceptible to metallic stent-related artefacts rendering interpretation of stented regions impossible
Copyright American Society of Nuclear Cardiology
